# Supplementary material for: Roles of Small Polyetherimide Moieties on Thermal Stability and Fracture Toughness of Epoxy Blends
Source: Polymers (Basel). 2021 Sep 28;13(19):3310. doi: 10.3390/polym13193310 (PMC8512680; doi:10.3390/polym13193310)
Supplement: Supplementary file 1 [file polymers-13-03310-s001.zip › polymers-1384286-supplementary.pdf]

polymers

MDPI

Supporting Information

Roles of Small Polyetherimide Moieties on Thermal Stability and Fracture Toughness of Epoxy Blends

Seul-Yi Lee [1], Min-Joo Kang [1], Seong-Hwang Kim [1], Kyong Yop Rhee [2,*], Jong-Hoon Lee [1] and Soo-Jin Park [1,*]

1 Department of Chemistry, Inha University, 100 Inharo, Incheon 22212, Korea; leesy1019@inha.ac.kr (S.-Y.L.); sa-rahlunar@naver.com (M.-J.K.); seonghwangkim@inha.edu (S.-H.K.); boy834@naver.com (J.-H.L.)

2 Department of Mechanical Engineering, College of Engineering, Kyung Hee University, 1732 Deogyeong-daero, Yongin 17104, Korea

* Correspondence: rheeky@khu.ac.kr (K.Y.R.), sjpark@inha.ac.kr (S.-J.P.); Tel.: +82-31-201-2565 (K.Y.R.); +82-32-876-7234 (S.-J.P.)

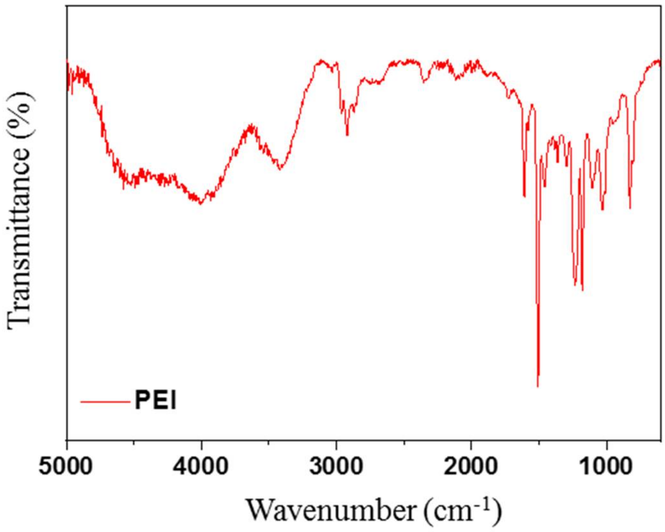

Figure S1. FT-IR spectrum of PEI.
